# Dual role of phosphatidylserine and its receptors in osteoclastogenesis

**DOI:** 10.1038/s41419-020-2712-9

**Published:** 2020-07-01

**Authors:** Jee-Hae Kang, Hyun-Mi Ko, Geum-Dong Han, Su-Young Lee, Jung-Sun Moon, Min-Seok Kim, Jeong-Tae Koh, Sun-Hun Kim

**Affiliations:** Dental Science Research Institute, School of Dentistry, Chonnam National University, Gwangju, 61186 Korea

**Keywords:** Cell biology, Molecular biology

## Abstract

Fusion and apoptosis share a breakdown of the membrane phospholipids asymmetry, modes of which are largely unknown in osteoclastogenesis. Here, we investigated the externalization of phosphatidylserine (PS) and its receptors, and their biological functions in osteoclastogenesis. Strong immunoreactivities in vivo for the PS receptors TIM4, BAI1, and STAB2 were observed in the TRAP-positive multinucleated cells in the alveolar bone that was being remodeled around the developing dental follicles in rats. These receptors were significantly upregulated during M-CSF/RANKL-induced in vitro osteoclastogenesis using mouse bone marrow-derived cells. PS externalization in preosteoclasts was increased by the M-CSF/RANKL treatment. Multinucleation of preosteoclasts was markedly inhibited by antibodies against PS and its receptors. Among the investigated lipid transporter proteins, floppases (*Abcb4*, *Abcc5*, and *Abcg1*) were upregulated, whereas flippases (*Atp11c* and *Atp8a1*) downregulated during osteoclastogenesis. Preosteoclast fusion was markedly blocked by the ATPase inhibitor Na_3_VO_4_ and siRNAs against *Abcc5* and *Abcg1*, revealing the importance of these lipid transporters in PS externalization. Further, the levels of *Cd47* and *Cd31*, don’t-eat-me signal inducers, were increased or sustained in the early phase of osteoclastogenesis, whereas those of *AnnexinI* and *Mfg-e8*, eat-me signals inducers, were increased in the late apoptotic phase. In addition, Z-VAD-FMK, a pan caspase inhibitor, had no effect on preosteoclast fusion in the early phase of osteoclastogenesis, whereas Abs against PS, TIM4, and BAI1 decreased osteoclast apoptosis during the late phase. These results suggest that PS externalization is essential for the whole process of osteoclastogenesis and share PS receptors and transporters in the early stage fusion and late stage apoptosis. Therefore, modulation of PS and its receptors could be a useful strategy to develop anti-bone resorptive agents.

## Introduction

Osteoclasts, important for the remodeling of the skeleton, are multinucleated tartrate-resistant acid phosphatase (TRAP)-positive bone-resorbing cells. The hematopoietic monocyte/macrophage precursors differentiate into osteoclasts by treatment of macrophage colony-stimulating factor (M-CSF) and receptor activator of nuclear factor-κB ligand (RANKL), two essential cytokines for osteoclast formation. During osteoclastogenesis, M-CSF/RANKL induces a master transcription factor, nuclear factor of activated T cells c1 (NFATc1), which increases the expression of osteoclast-specific genes such as *Acp5* (encoding TRAP) and *Cathepsin K*^1^. Lifespan of osteoclasts from the fusion of mononucleated precursors to apoptotic death is 1–2 weeks and quickly removed by phagocytes, while that of osteoblasts is about 3 months^2,3^. The brief lifespan of osteoclasts suggests a common mechanism that conveys signals leading to apoptotic death soon after fusion for multinucleation. In fact, a breakdown of the membrane phospholipids asymmetry is detected in both apoptosis and cell fusion, suggesting that understanding of underlying mechanism of phospholipids exposure during osteoclastogenesis is necessary to develop bone resorptive/antiresorptive agents.

Cellular fusion is a common physiological phenomenon with broad biological significance in fertilization, formation of skeletal myofibers and macrophage giant cells, and osteoclastogenesis^4^. During fusion, the composition and distribution of the phospholipids undergoes dynamic changes, leading to alterations in the stability and fluidity of the bilayer^5^. Exposure of phosphatidylserine (PS), a phospholipid component of the cell membrane, on the surface of myoblasts regulates cell fusion and blockade of PS externalized on the cell surface inhibits syncytium formation in macrophages, trophoblasts, and myoblasts, indicating that PS has important functions in cell fusion^6–9^.

It is known that PS exposure on the cell surface is accomplished by the inactivation of flippases, which transport PS from the outer leaflet to the cytoplasmic surface of the plasma membrane^10^. Activation of Ca^2+^-dependent ATP-independent scramblases, such as XKR8 and TMEM16F, and ATP-dependent transporter floppase ABCA1 also contributes to PS exposure and asymmetry^11–13^. Although the essential functions of these transporters in the regulation of plasma membrane phospholipid asymmetry in response to physiological and pathological conditions are well established, their roles in PS exposure during osteoclastogenesis are largely unknown.

Apoptosis, essential for tissue homeostasis and immune tolerance, is stimulated by the intrinsic and extrinsic signaling pathways which induce a series of events such as caspases activation, cytochrome C release, DNA fragmentation, and PS externalization followed by elimination of the target cell. Exposed PS on the cell surface during apoptosis functions as an eat-me signal to phagocytic cells leading apoptotic cell clearance^14^. PS regulates cell-to-cell fusion and apoptosis by binding to soluble proteins, such as AnnexinV and lactadherin, or by engaging the PS receptors^15–17^. Over a dozen PS receptors have been identified, and their functions including efferocytosis, immune suppression and tolerance, viral infection, and apoptosis have been determined^14^. Recent studies showed that the recognition of cell surface PS by receptors such as BAI1 and stabilin2 (STAB2) is involved in both apoptotic cell clearance and myoblast fusion^6,18^. Osteoclasts, monocytes/macrophages, and dendritic cells express the PS receptors TIM1, TIM4, BAI1, and STAB2^14^, strongly implying that these receptors may play a role in the apoptotic death as well as fusion of osteoclasts.

Although the expression and activities of PS receptors in nonskeletal cells have been determined, those in osteoclastogenesis are currently largely unknown. Further, the externalization mechanism and roles of PS in the fusion and subsequent postfusion apoptosis of osteoclasts are elusive. We assessed biological roles of PS and its receptors in the regulation of preosteoclast fusion and apoptosis, both of which may share PS machineries, considering the brief lifespan of osteoclasts. Inhibition of fusion and concurrent stimulation of apoptosis is needed to inhibit osteoclastogenesis. Here, we report common action mechanism and different modalities of PS machineries in the fusion and apoptotic process of osteoclasts.

## Materials and methods

### Animals, isolation of mouse bone marrow-derived cells (BMDCs) and osteoclast formation, and reagents

Animals were housed in a controlled environment. All experimental procedures were conducted in accordance with the guidelines of the Chonnam National University Institutional Animal Care and Use Committee. Eight-week-old C57BL/6 male mice and 0-day-old Sprague-Dawley rats were randomly used.

Mouse bone marrow cells were harvested by flushing femurs and tibias with 10 ml of α-MEM (GIBCO BRL, Grand Island, NY, USA) supplemented with 10% fetal bovine serum (GIBCO BRL) and 1% antibiotic-antimycotic (AA; GIBCO BRL). Harvested cells from each individual mouse were used for each independent experiment. All cell experiments were used for data acquisition without preestablished criteria. Non-adherent cells were cultured with M-CSF (50 ng/ml; R&D Systems, Minneapolis, MN, USA) and RANKL (50 ng/ml; R&D Systems) for 3, 6, and 9 days. The days 0 to 6 and 6 through 9 of the culture were defined as the early and late phases of osteoclastogenesis, respectively. The culture medium was replaced with fresh medium every 3 days. After 3 days of culture, preosteoclasts (mononucleated TRAP-positive cells) were observed, and mature osteoclasts (multinucleated TRAP-positive cells, ≥3 nuclei) were gradually increased in the late phase of osteoclastogenesis. Osteoclast formation was imaged under a light microscope. In this study, BMDC referred to the non-adherent BMDCs.

Pan caspase inhibitor, Z-VAD-FMK was purchased from R&D Systems, dissolved in DMSO (Sigma-Aldrich Co., St. Louis, MO, USA). An ATPase inhibitor, NA_3_VO_4_, was purchased from Sigma-Aldrich. Normal mouse, rabbit, and rat IgG used as controls were purchased from ThermoFisher Scientific (Rockford, IL, USA, Cat. no. 31903, 31235, and 31933).

### Immunofluorescence staining

Alveolar bone containing the maxillary molar germs was isolated on postnatal day 0 and fixed with a 3.7% paraformaldehyde solution. Tissues were decalcified and paraffin-embedded. Cultured osteoclasts were fixed in 3.7% paraformaldehyde. Immunofluorescence staining was performed with a TSA™ kit (Invitrogen, Carlsbad, CA, USA). To detect the expression of PS receptors, cells and tissues were incubated with primary antibodies (Abs) against TIM4 (LifeSpan BioSciences Inc., Seattle, WA, USA, Cat. no. LS-B598), BAI1 (ThermoFisher Scientific, Cat. no. PA1-46465), and STAB2 (Medical & Biological Laboratories, Nagoya, Japan, Cat. no. D317-3), and then with biotinylated secondary Ab. Finally, these were counterstained with propidium iodide (PI; Invitrogen), and examined with an LSM confocal microscope (Carl Zeiss, Göttingen, Germany).

### TRAP staining

Osteoclasts were fixed and incubated with a mixture of the solutions fast Garnet GBC, sodium nitrite, naphthol AS-BI phosphoric acid, acetate, and tartrate in the leukocyte acid phosphatase assay kit (Sigma-Aldrich, Cat. no. 387A). Osteoclast formation was imaged under a light microscope. The size of each osteoclast and the number of nuclei were determined by an image analysis software (iSolution FL Auto; IMT i-Solution Inc., Burnaby, BC, Canada).

### PS externalization assay

BMDCs were cultured for 6 days in α-MEM containing 10% FBS, 1% AA, and 50 ng/ml M-CSF with or without 50 ng/ml RANKL. Subsequently, live cells were incubated with a PS Ab (Millipore Corporation, Bedford, MA, USA, Cat. no. 05-719), and immunofluorescence stained. Externalized PS in live cells was assayed with AnnexinV-FITC (BioLegend, San Diego, CA, USA, Cat. no. 640906). The cells were counterstained with PI or DAPI and then examined with an LSM confocal microscope or Lionheart FX automated microscope (BioTek, Winooski, VT, USA).

### Quantitative real-time reverse transcription polymerase chain reaction (RT-PCR)

Total RNA was extracted with TRIzol Reagent (Invitrogen). Real-time amplification of cDNA was conducted in a Rotor-Gene 3000 System (Corbett Research, Morklake, Australia) using the SYBR Green PCR Master Mix Reagent Kit (Qiagen, Valencia, CA, USA). The primers used were shown in Table 1. The mean cycle threshold (Ct) values from triplicate measurements were used to calculate gene expression, and β-actin was employed as an internal control for normalization. Relative gene expression was calculated using Corbett Robotics Rotor-Gene software (Rotor-Gene 6 version 6.1, Build 90).

**Table 1:** Oligonucleotides used for RT-PCR.

Target gene (NCBI-accession number)	Oligonucleotide sequence
*Tim4* (NM_178759.4)	5′-GTGGGTCCTGTCTGGTTGTAT-3′ (forward) 5′-ACTGACAGTGTTCAAGCCCA-3′ (reverse)
*Bai1* (NM_174991.3)	5′-TGAAGTGCCGTGTGGTAGAC-3′ (forward) 5′-GCACTGATCTACAGGCCAGA-3′ (reverse)
*Stab2* (NM_138673.3)	5′-CACTATGTCGGGGATGGACG-3′ (forward) 5′-GGGAGCGTAGGTGGAATACG-3′ (reverse)
*Nfatc1* (NM_016791)	5′-TCATCCTGTCCAACACCAAA-3′ (forward) 5′-TCACCCTGGTGTTCTTCCTC-3′ (reverse)
*Acp5* (NM_007388)	5′-CAGCAGCCAAGGAGGACTAC-3′ (forward) 5′-ACATAGCCCACACCGTTCTC-3′ (reverse)
*Cd31* (NM_008816.3)	5′-AAGCAGCACTCTTGCAGTCA-3′ (forward) 5′-CATCTCCACGGGTTTCTGTT-3′ (reverse)
*Cd47* (NM_010581.3)	5′-CGATGCCATGGTGGGAAACT-3′ (forward) 5′-ACCTCCTTTCTCCTCCTCGT-3′ (reverse)
*AnnexinI* (NM_010730.2)	5′-AGGAAAGTTGCTTTGGCAGA-3′ (forward) 5′-TGACTTGCTTATGGGGCTTT-3′ (reverse)
*Mfg*-e8 (NM_008594.2)	5′-AGAGTTGCCCTTCACCCTTT-3′ (forward) 5′-TTGGGGTGGTTGGTACAGAT-3′ (reverse)
*Atp8a1* (NM_009727.3)	5′-AGAAATGGTGCATGGGAAAT-3′ (forward) 5′-CCTTCACTATCTCCCCCACTG-3′ (reverse)
*Atp11c* (NM_001037863.2)	5′-AGTTGTAAAGAATGTTCGAAGAAGAA-3′ (forward) 5′-TCAGATGCCCTTCTACAGCTC-3′ (reverse)
*Abcb4* (NM_008830.2)	5′-CGACTTTGAACTAGGCAGCA-3′ (forward) 5′-AACAGGCCAATTAAATTCACTTTC-3′ (reverse)
*Abcc5* (NM_013790.2)	5′-GTTCTGGGCTCTGACAGGAT-3′ (forward) 5′-GACCGATGGGGTGTCAAA-3′ (reverse)
*Abcg1* (NM_009593.2)	5′-GGGTCTGAACTGCCCTACCT-3′ (forward) 5′-TACTCCCCTGATGCCACTTC-3′ (reverse)
*β-actin* (NM_007393.5)	5′-GATCTGGCACCACACCTTCT-3′ (forward) 5′-GGGGTGTTGAAGGTCTCAAA-3′ (reverse)

### Western blot analysis

Protein extracts were prepared using CytoBuster Protein Extraction Reagent (Novagen, Madison, WI, USA), separated by SDS-polyacrylamide gel electrophoresis, and transferred to a Protran nitrocellulose membrane (Whatman GmbH, Dassel, Germany). The membrane was incubated with Abs against TIM4, BAI1, or STAB2. A purified mouse monoclonal primary Ab against β-ACTIN (ThermoFisher Scientific, Cat. no. MA5-15739) was used as a control. The blots were incubated with horseradish peroxidase-conjugated anti-rabbit or anti-mouse IgG (Cell Signaling Technology, Danvers, MA, USA, Cat. no. 7074 and 7076), developed with the HRP Substrate Luminol Reagent (Millipore Corporation, Billerica, MA, USA) and subsequently photographed using an LAS4000 loaded with ImageReader LAS-4000 software (Fujifilm, Minatoku, Tokyo, Japan). The relative level of each protein was quantified with Scion Image software (Scion, Frederick, MD, USA).

### Small interfering RNA (siRNA) transfection

To knockdown floppase expression, osteoclast precursors were transfected with siRNAs against *Abcb4*, *Abcc5*, and *Abcg1* or a scrambled control siRNA (Invitrogen) using Lipofectamine RNAiMAX transfection reagent (Invitrogen). One day after osteoclastogenesis induction, the cells were treated with the siRNAs (10 μM). The expression levels of Nfatc1 and each siRNA-knocked down gene were determined by real-time RT-PCR on day 4, and TRAP staining was performed on day 6 after treatment with M-CSF/RANKL.

### TUNEL assay

A TUNEL staining assay was performed in osteoclasts with the DeadEnd^TM^ Colorimetric TUNEL System (Promega) and visualized with a DM microscope (Leica, Wetzlar, Germany).

### Statistical analysis

Statistical significance was assessed by Student’s *t* test and two-way ANOVA using GraphPad Prism 5 software. *p* value < 0.05 was considered significant. The results are shown as the mean ± SEM of triplicate experiments. Reproducible results were obtained, and representative data are shown in the figures.

## Results

### PS receptors are expressed in TRAP-positive multinucleated cells

**Fig. 1:**
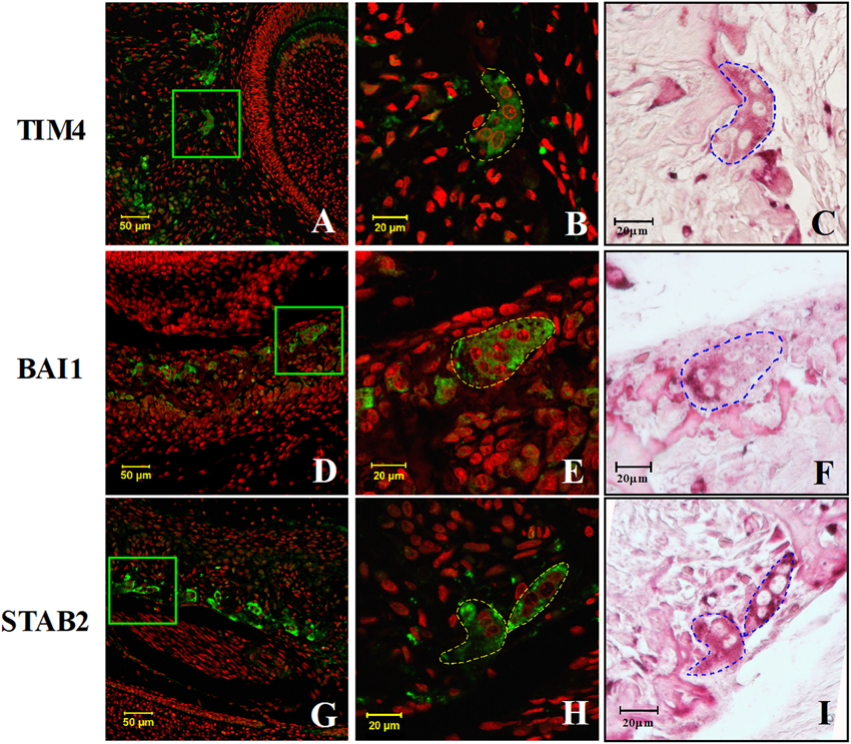
PS receptors are expressed in TRAP-positive multinucleated cells within the developing tooth germ of the rat alveolar bone. Three alveolar bone tissues on postnatal day 0 were decalcified, formalin-fixed, paraffin-embedded, and serial sectioned. Tissue sections were immunofluorescence stained with Abs against the PS receptors TIM4, BAI1, and STAB2 (**a**, **d**, and **g**). Representatively stained results were exhibited in figures. The areas in the green boxes are magnified (**b**, **e**, and **h**), and the same sections were stained for TRAP (**c**, **f**, and **i**). The yellow and blue dotted lines show the same cell.

**Fig. 2:**
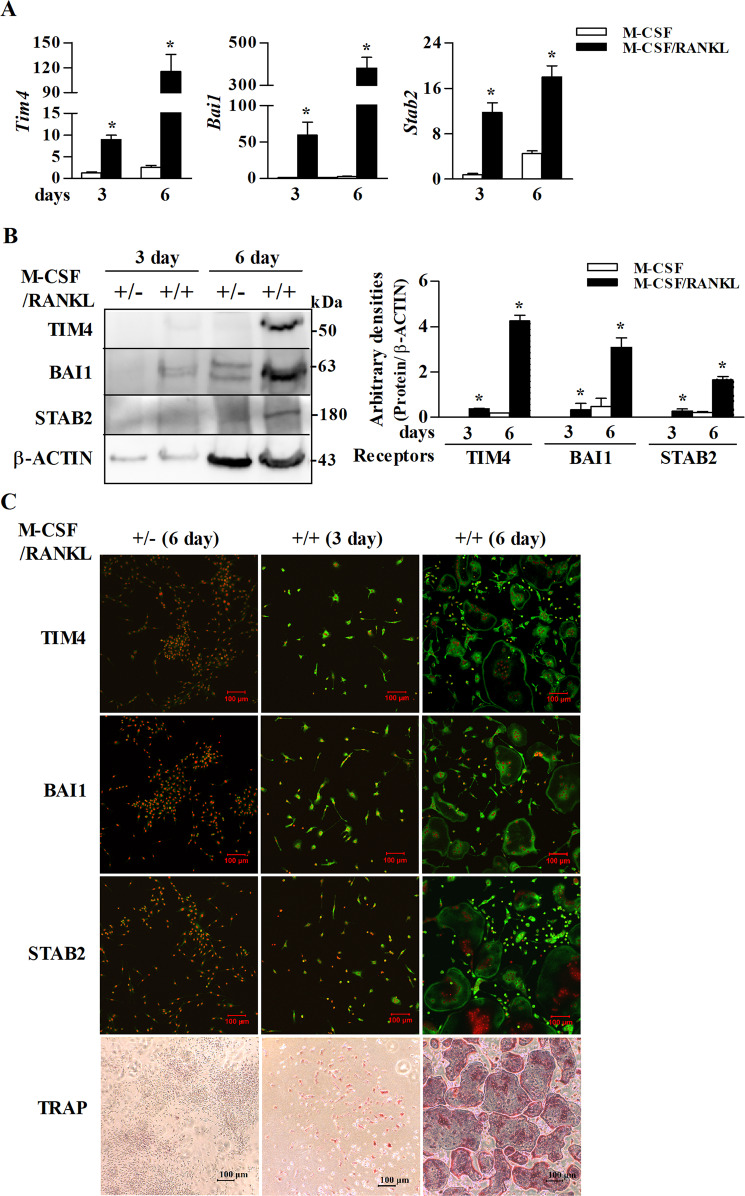
PS receptor levels progressively increase during osteoclastogenesis. The mRNA and protein levels of the PS receptors in BMDCs cultured for 3 and 6 days in the presence of M-CSF or M-CSF/RANKL were determined by real-time RT-PCR (**a**) and western blot analyses (**b**), respectively. The relative protein levels of the PS receptors were quantified and normalized to the levels of β-ACTIN. **c** Formalin-fixed cells were immunofluorescence stained with Abs against each PS receptor on days 3 and 6 after treatment with M-CSF or M-CSF/RANKL, and stained for TRAP. The data (mean ± SEM) were acquired from three independent experiments. *p < 0.01, compared with the M-CSF-treated group.

First, we performed an immunohistological analysis to access PS receptors in vivo. Interestingly, TIM4, BAI1, and STAB2 were strongly expressed in TRAP-positive multinucleated cells in the alveolar bone that was being massively remodeled around the developing dental follicles (Fig. 1). To confirm the expression of the PS receptors in osteoclasts during osteoclastogenesis in vitro, BMDCs were cultured. As shown in Fig. 2a, b, the mRNA and protein levels of the PS receptors in BMDCs treated with M-CSF/RANKL were markedly higher than those treated with M-CSF alone. The immunofluorescence staining clearly demonstrated that these receptors were highly expressed in the mononucleated and multinucleated osteoclasts (Fig. 2c). In contrast, the receptors could not be detected in the M-CSF-treated BMDCs on day 3, and only weak expression was observed on day 6 by western blotting and immunofluorescence staining (Fig. 2b, c).

### PS and PS receptors play critical roles in the fusion of preosteoclasts

**Fig. 3:**
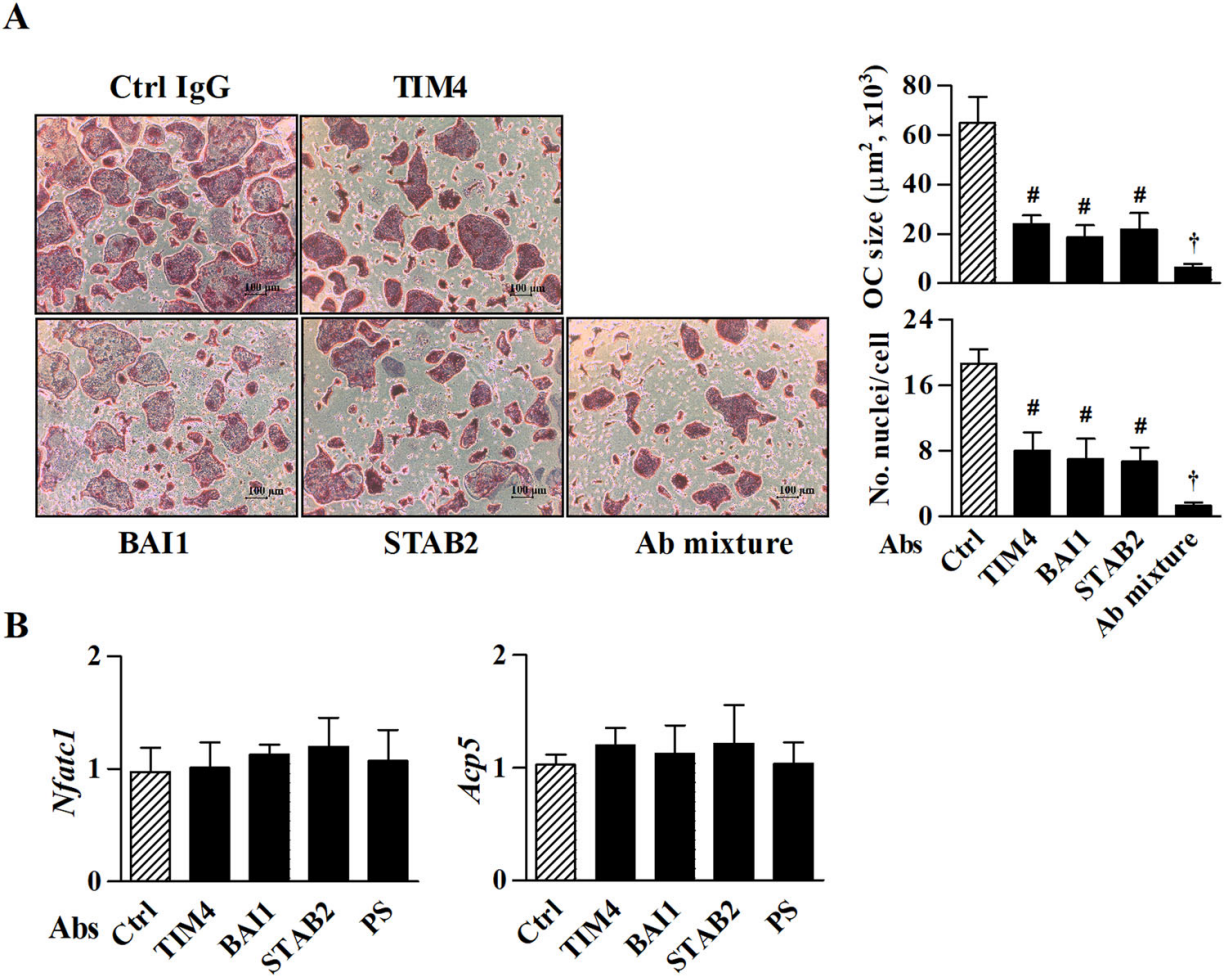
PS receptors are cooperatively involved in the fusion of preosteoclasts. **a** BMDCs cultured in the presence of M-CSF/RANKL were treated with Abs (0.5 μg/ml) against each PS receptor, Ctrl IgG, or a mixture of all Abs against the PS receptors TIM4, BAI1, and STAB2, which was used to probe their synergistic effects. Normal rabbit and rat IgG were used as controls. Osteoclasts were stained for TRAP on day 6 of M-CSF/RANKL treatment. The size of each osteoclast was determined, and the number of nuclei was counted. **b** *Nfatc1* and *Acp5* mRNA levels were analyzed by real-time RT-PCR on day 6 after treatment M-CSF or M-CSF/RANKL. The data (mean ± SEM) were acquired from three independent experiments. ^#^*p* < 0.01, compared with the Ctrl IgG-treated group; ^†^*p* < 0.05, compared with the PS receptor Ab-treated group. Ab antibody. Ctrl control.

Next, we determined the involvement of PS receptors in the fusion of preosteoclasts using Abs against TIM4, BAI1, and STAB2. As shown in Fig. 3a, the level of preosteoclast fusion induced by M-CSF/RANKL for 6 days was dramatically decreased by each PS receptor Abs but not by the control IgG, and inhibited to a greater extent by a mixture of the Abs than by each Ab alone. These results suggest that these PS receptors synergistically induce the multinucleation of preosteoclasts. However, Abs against PS and PS receptors had no significant effects on M-CSF/RANKL-induced *Nfatc1* and *Acp5* expression during osteoclast differentiation and function (Fig. 3b), indicating that osteoclastic commitment was not affected by the interaction between PS and PS receptors.

**Fig. 4:**
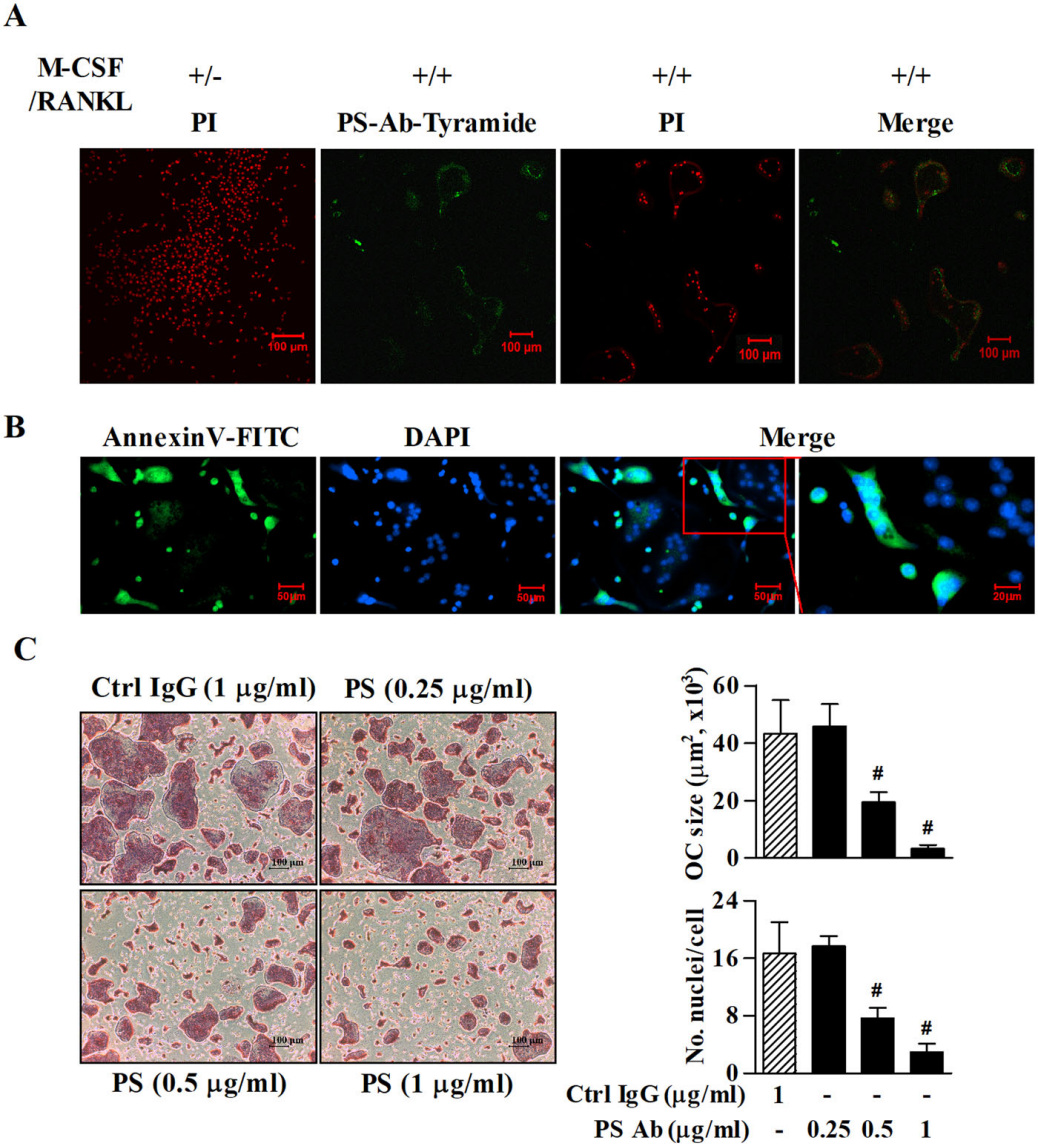
PS externalization in preosteoclasts during osteoclastogenesis plays a critical role in the fusion of preosteoclasts. **a** BMDCs were cultured in the presence of M-CSF or M-CSF/RANKL. Then, the live cells were immunofluorescence stained with Ab against PS on day 4 of treatment. After formalin-fixation, the nuclei were counterstained with PI. **b** Live BMDCs cultured for 4 days with M-CSF/RANKL were stained with AnnexinV-FITC. After formalin-fixation, the nuclei were counterstained with DAPI. The area shown in the red box is magnified. **c** BMDCs were treated with the indicated concentrations of PS-neutralizing Ab or Ctrl Ab for 6 days in the presence of M-CSF/RANKL. Normal mouse IgG was used as a control. The size of each osteoclast was determined, and the number of nuclei was counted. The data (mean ± SEM) were acquired from three independent experiments. ^#^*p* < 0.01, compared with the Ctrl IgG-treated group. Ab antibody. Ctrl control.

To evaluate the localization and function of PS during osteoclastogenesis, we visualized and blocked externalized PS with a neutralizing Ab or the PS-binding protein, AnnexinV labeled with FITC. Live multinucleated osteoclasts induced by M-CSF/RANKL were immunofluorescence stained with a PS Ab on day 4 of treatment and a notable fluorescence signal was observed on the outer side of the cell membrane (Fig. 4a). Similarly, mononucleated and small multinucleated osteoclasts in the process of fusing with large multinucleated osteoclasts were more strongly stained with AnnexinV-FITC (Fig. 4b). However, no fluorescence signal was detected in BMDCs treated with M-CSF alone (data not shown). Furthermore, neutralizing Ab against PS but not control IgG significantly inhibited preosteoclast multinucleation in a dose-dependent manner (Fig. 4c). Taken together, the above results reveal the importance of PS and PS receptors in preosteoclast fusion.

### PS externalization is important for not only preosteoclast fusion but also osteoclast apoptosis

**Fig. 5:**
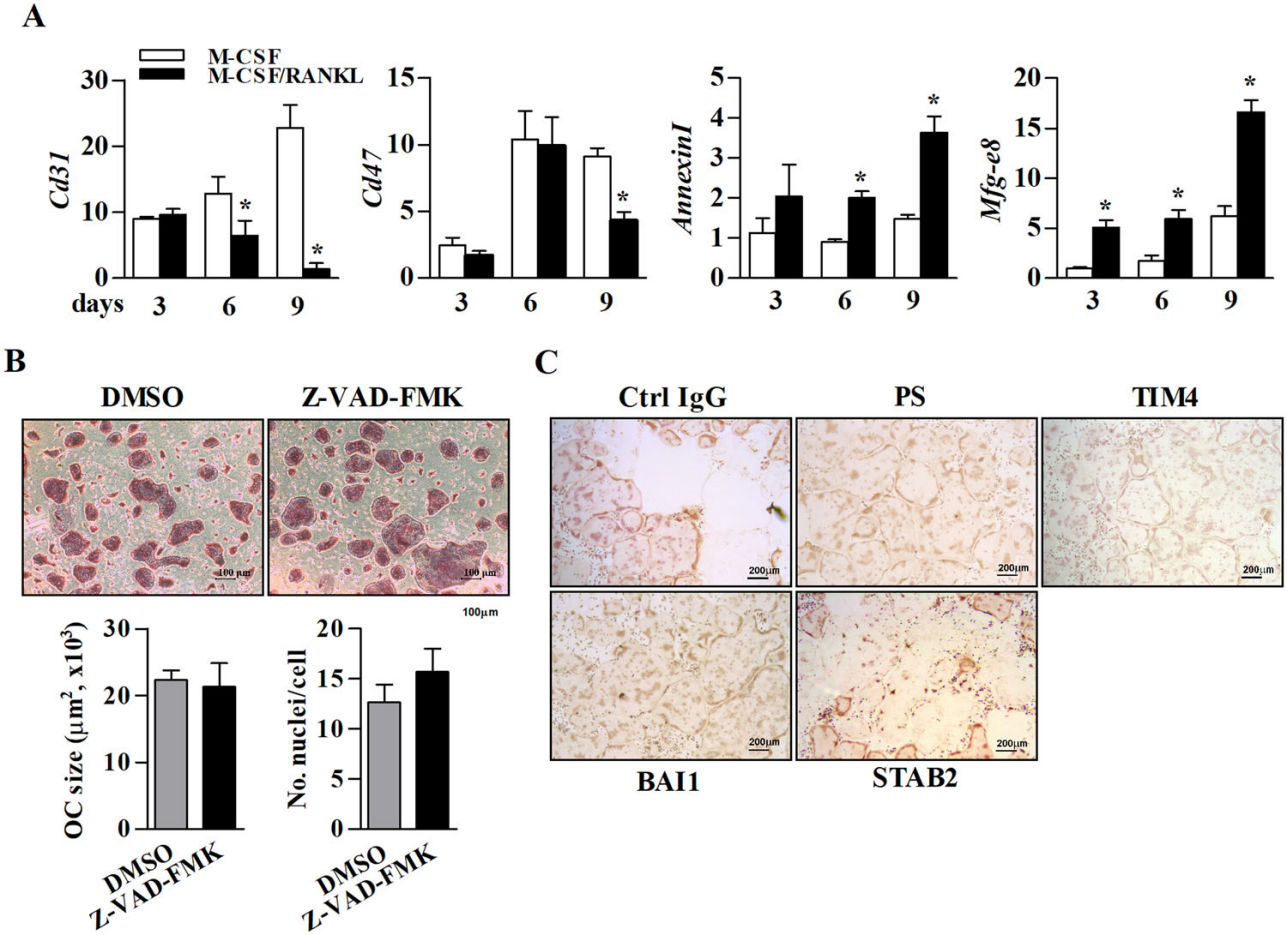
Externalized PS is involved in the multinucleation and apoptosis of osteoclasts in the early and late phases of osteoclastogenesis, respectively. **a** The mRNA levels of *Cd31*, *Cd47*, *AnnexinI*, and *Mfg-e8* in BMDCs cultured in the presence of M-CSF or M-CSF/RANKL for 3, 6, and 9 days were determined by real-time RT-PCR. **b** Cells cultured in the presence of M-CSF/RANKL were treated with the pan caspase inhibitor, 50 μM Z-VAD-FMK, or 0.25% DMSO as a control and then stained for TRAP on day 6. The size of each osteoclast was determined, and the number of nuclei was counted. **c** Cells cultured in the presence of M-CSF/RANKL were treated with Abs (0.5 μg/ml) against PS and each PS receptor or Ctrl IgG on day 5, and TUNEL assay was performed on day 9. Normal rabbit and rat IgG were used as controls. The data (mean ± SEM) were acquired from three independent experiments. *p < 0.01, compared with the M-CSF-treated group. Ctrl control.

In mammalian cells, the role of externalized PS during apoptosis is well-understood^16^. To investigate whether externalized PS during osteoclastogenesis is involved in osteoclast apoptosis, we first determined the expression profile of *Cd31*, *Cd47*, *AnnexinI*, and *Mfg-e8* during the course of osteoclastogenesis. As shown in Fig. 5a, *Cd47*, which induces a don’t-eat-me signal through its interaction with SIRPα and promotes preosteoclast fusion in the early stage of fusion^19,20^, was steadily increased until day 6 and was clearly inhibited on day 9 after treatment with M-CSF/RANKL. In addition, *Cd31*, another don’t-eat-me signal, was gradually decreased from day 6 to day 9 in the M-CSF/RANKL-treated group. In contrast, *AnnexinI* and *Mfg-e8*, which facilitate engulfment by binding to PS and inhibits osteoclastogenesis^19,21^, were markedly increased on day 9 compared with the levels in the M-CSF only-treated group, implying that externalized PS may be involved in apoptosis during the late phase of osteoclastogenesis. Furthermore, to double-check that externalized PS is irrelevant with apoptosis induction in osteoclasts during the early phase of osteoclastogenesis, if any, we examined the effect of the pan caspase inhibitor Z-VAD-FMK on the formation of multinucleated osteoclasts. As shown in Fig. 5b, Z-VAD-FMK had no effect on the formation of multinucleated osteoclasts induced by M-CSF/RANKL. Our previous study showed increased apoptosis in mature osteoclasts in the late stage of M-CSF/RANKL-induced osteoclastogenesis in BMDCs^3^. Therefore, to confirm the role of PS and its receptors in osteoclast apoptosis in the late phase, we blocked each receptor with an Ab. Expectedly, apoptosis during the late phase of osteoclastogenesis on day 9 was decreased by treatment with Abs against PS, TIM4, and BAI1. However, the control Ab and the STAB2 Ab had no effects on osteoclast apoptosis in the late stage of osteoclastogenesis (Fig. 5c). Collectively, these results suggested that non-apoptotic PS externalization during the early stage of osteoclastogenesis is essential for M-CSF/RANKL-induced fusion of preosteoclasts but not for induction of apoptosis. In addition, externalized PS and the PS receptors TIM4 and BAI1, but not STAB2, play a pivotal role in the apoptosis of osteoclasts in the late phase of osteoclastogenesis.

### ATP-dependent lipid transporters *Atp11c*, *Abcc5*, and *Abcg1* contribute to PS externalization, leading to osteoclast fusion and apoptosis

**Fig. 6:**
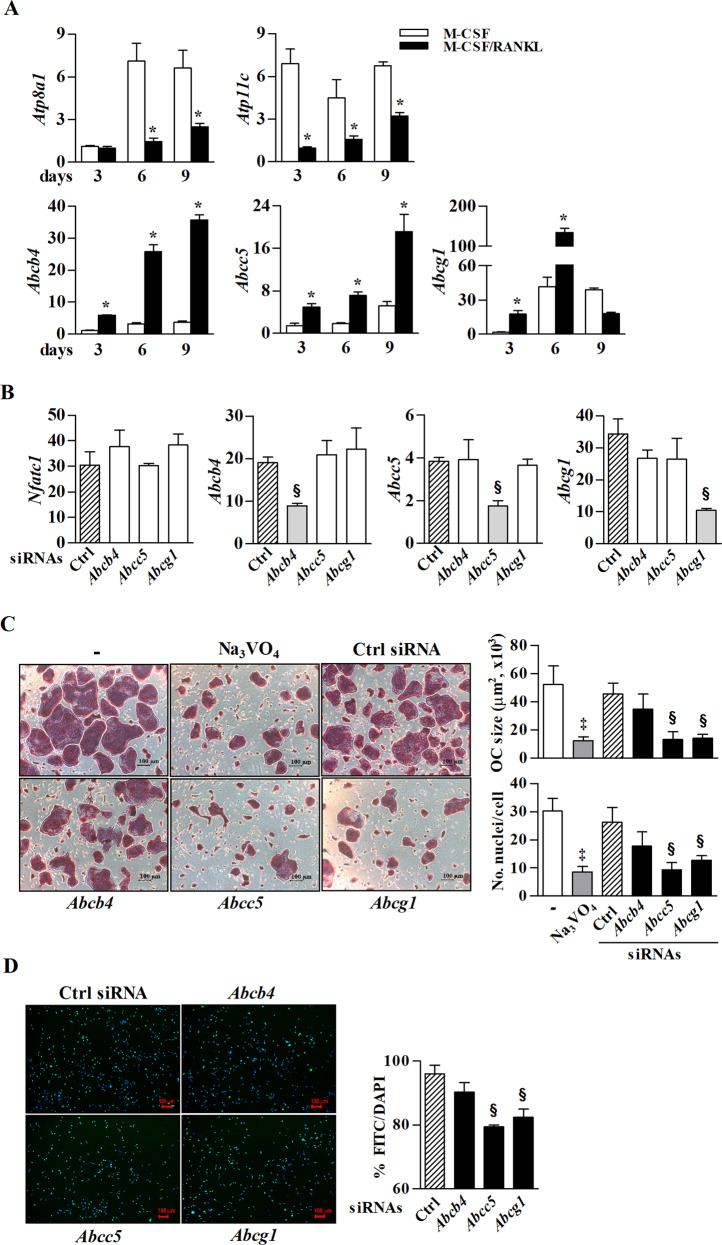
ATP-dependent transporters contribute to PS externalization and preosteoclast fusion during osteoclastogenesis. **a** The mRNA levels of ATP-dependent transporters were analyzed in BMDCs treated with M-CSF or M-CSF/RANKL on days 3, 6, and 9 by real-time RT-PCR. **b** Cells were treated with siRNAs against the transporters *Abcb4*, *Abcc5*, and *Abcg1* for 3 h. Expression of the osteoclast-related gene *Nfatc1* and the efficiency of siRNA-mediated knockdown was validated by real-time RT-PCR on days 6 and 4 after treatment with M-CSF/RANKL, respectively. **c** BMDCs cultured in the presence of M-CSF/RANKL were treated with the ATPase inhibitor, NA_3_VO_4_ (10 μM), or siRNAs against *Abcb4*, *Abcc5*, and *Abcg1* (10 nM), and then TRAP staining was performed on day 6. The size of each osteoclast was analyzed, and the number of nuclei was counted. **d** Live osteoclast precursors treated with specific siRNAs against *Abcb4*, *Abcc5*, and *Abcg1* or Ctrl siRNA in the presence of M-CSF/RANKL were stained with AnnexinV-FITC on day 3. After formalin-fixation, the nuclei were counterstained with DAPI. The density of FITC/DAPI staining was analyzed by a Lionheart FX automated microscope using Gen5^TM^ software. The data (mean ± SEM) were acquired from three independent experiments. *p < 0.01, compared with the M-CSF-treated group; ^§^*p* < 0.01, compared with the Ctrl siRNA-treated group; ^‡^*p* < 0.01, compared with the M-CSF/RANKL-treated group. Ctrl control.

The abundance and distribution of PS on the cell surface are modulated by ATP-dependent and -independent transporters^22^. Although flippases and floppases, two classes of ATP-dependent lipid transporters are well known, their roles in PS exposure on the cell surface during osteoclastogenesis have been hardly studied. Therefore, we examined the expression of 52 floppases and 10 flippases to determine the molecules responsible for PS externalization during osteoclastogenesis, by real-time RT-PCR (data not shown). Among them, flippases *Atp8a1* and *Atp11c* were decreased in osteoclastogenesis with the significant decrease of the flippase *Atp11c* in the early phase (Fig. 6a). Of those of the screened floppases, the mRNA expression levels of *Abcb4* and *Abcc5* were gradually increased from day 3 to day 9 of M-CSF/RANKL treatment, and that of *Abcg1* mRNA significantly increased from day 3 to day 6 (Fig. 6a). To further investigate the effect of the floppases, whose expression was markedly increased, on PS externalization and preosteoclast fusion, we knocked down the expression of *Abcb4*, *Abcc5*, and *Abcg1* using specific siRNAs. These siRNAs treatment did not lead to a discernible difference in M-CSF/RANKL-induced *Nfatc1* expression (Fig. 6b). Interestingly, M-CSF/RANKL-induced preosteoclast fusion was decreased by treatment with NA_3_VO_4_, an ATPase inhibitor, as well as the *Abcc5* and *Abcg1* siRNAs. In addition, PS externalization was significantly inhibited by the *Abcc5* and *Abcg1* siRNAs. However, the *Abcb4* and control siRNAs did not have any effects on PS externalization and preosteoclast fusion during osteoclastogenesis (Fig. 6c, d). These results suggest that the flippase (*Atp8a1* and *Atp11c*) and the floppases (*Abcc5* and *Abcg1*) may be responsible for PS-mediated cellular fusion (early stage) and apoptosis (late stage) in osteoclastogenesis.

## Discussion

In this study, we for the first time demonstrated the mechanism of PS externalization, its modes of action via PS receptors, and its shared role in preosteoclast fusion and apoptosis of matured osteoclasts. Studies have demonstrated that PS and its receptors are involved in apoptosis and cell-to-cell fusion^16,18,23,24^. The essential activity of PS in cell-to-cell fusion is regulated by PS-binding molecules, such as lactadherin, annexins, and PS receptors^15–17^. BAI1 and STAB2 strengthen the effects of PS exposure on the outer membrane during myoblast fusion^6,23^. Recently, it has been reported that PS and phosphatidylethanolamine on the cell surface are essential for osteoclast fusion^25,26^.

TIM4, BAI1, and STAB2 have been identified as mediators of apoptotic and necrotic cell clearance^19^. These PS receptors are highly expressed in osteoclasts, which effectively phagocytose apoptotic cells^18^. In this study, we in vivo revealed the expression of TIM4, BAI1, and STAB2 in TRAP-positive multinucleated cells in the alveolar bone that was undergoing active remodeling. Furthermore, the expression levels of these in preosteoclasts gradually increased in the early phase of osteoclastogenesis. During myogenic differentiation, STAB2 expression in myoblasts is upregulated and contributes to PS-dependent cell fusion, while TIM4 and BAI1 remain at basal expression levels. However, activation of BAI1 by apoptotic myoblasts has been shown to upregulate the fusion of healthy myoblasts^23^. Consistent with these results, we demonstrated the role of PS and PS receptors in cell-to-cell fusion by treating cells with Abs against PS and PS receptors in the early osteoclastogenesis phase. Moreover, we demonstrated that PS receptors either worked alone or in cooperation to promote the preosteoclast fusion.

Efficient clearance of osteoclasts is crucial for bone remodeling. As seen in most mammalian cells, PS is externalized obviously to outer leaflet of cell membrane during apoptosis of osteoclasts^25^. PS, which is an eat-me signal displayed on the outer membrane of apoptotic cells, binds to its receptors on phagocytes to stimulate efferocytosis. In addition to directly interacting with its receptors, PS also augments efferocytosis by bridging molecules such as AnnexinI and MFG-E8, eat-me signals^19^. In contrast, CD31 and CD47, don’t-eat-me signals, block the engulfment of apoptotic cells^19,20^. These findings suggest that the functions of PS are dependent on membrane-anchored signal molecules and soluble bridging molecules that regulate diverse intracellular events. In this study, we evaluated the expression of extracellular players that regulate apoptotic clearance during osteoclastogenesis. *Cd31* and *Cd47*, which are required for preosteoclast fusion to differentiate functional osteoclasts, were expressed from the early phase and their levels gradually decreased in the late phase of osteoclastogenesis. In contrast, *AnnexinI* and *Mfg-e8* were markedly upregulated in the late phase of osteoclastogenesis. In addition, PS was externalized not only in the early phase for preosteoclast fusion but also in the late phase for the apoptosis of osteoclasts through its interaction with PS receptors during osteoclastogenesis. Taken together, our observations indicate that the two different functions of PS are thought to be mediated by membrane-anchored or bridging molecules that are expressed with PS.

The movement of PS to the outer leaflet of the cell membrane is related to Ca^2+^ influx, oxidative stress, and cytokines, and results from the (in)activation of lipid transporters and scramblases^13,16,22,27^. Flippases and floppases are ATP-dependent lipid transporters that are responsible for the asymmetric distribution of lipids in the membrane. Flippases are highly selective for PS and transport it cytofacially, whereas floppases are mainly nonspecific and function as exofacial transporters^10^. In immune cells, ATP11C transports PS cytofacially. Moreover, ABCC5 and ABCG1 have been reported to be involved in osteoclast formation and PS translocation, respectively^28–30^. In this study, we analyzed the expression of ATP-dependent transporters during osteoclastogenesis and found that the PS-specific transporter *Atp11c* was downregulated, but the exofacial transporters *Abcc5* and *Abcg1* were upregulated in the early phase of osteoclastogenesis, leading to PS externalization and subsequent preosteoclast fusion. The precise mechanisms of PS flopping catalyzed by ABCC5 and ABCG1 need to be investigated further.

In summary, we revealed PS externalization and the interaction of PS with its receptors in M-CSF/RANKL-induced preosteoclasts in vitro and bone-resorbing osteoclasts in vivo. In addition, the ATP-dependent lipid transporters contributed to PS externalization, which was needed for both early stage preosteoclast fusion and late stage matured osteoclast apoptotic death. Considering the brief lifespan of osteoclasts, blockade of preosteoclast fusion by masking the interaction between PS and PS receptors could be an effective strategy to develop anti-bone resorptive agents.
